# TDP‐43 proteinopathy induced transcriptional alterations in Alzheimer’s disease

**DOI:** 10.1002/alz.089794

**Published:** 2025-01-03

**Authors:** Ryan S Pevey

**Affiliations:** ^1^ Barrow Neurological Institute, Phoenix, AZ, USA; Arizona State University, Tempe, AZ USA

## Abstract

**Background:**

TDP‐43 is an RNA binding protein that is a pathological hallmark of multiple neurodegenerative diseases including Amyotrophic lateral sclerosis (ALS), frontotemporal dementia (FTD), and Alzheimer’s disease (AD). The frequency of observed TDP‐43 pathology is estimated at 97% in ALS, 45% in FTD and 40‐57% in AD and is characterized by a mislocalization of TDP‐43 from the nucleus to the cytoplasm. Indeed, TDP‐43 is the third most common proteinopathy in AD, behind only Amyloid beta and Tau. The normal function of TDP‐43 has been linked to multiple steps in RNA processing including transcription, splicing, RNA transport, stability, localization, and translation. Most studies aimed at understanding TDP‐43 pathology have been performed in the context of ALS and FTD, while little is known about the molecular mechanisms and consequences of TDP‐43 pathology in AD. We hypothesize that TDP‐43 dependent alterations in gene expression in neurons with TDP‐43 pathology are contributing to neuronal dysfunction and degeneration in AD and related dementias.

**Methods:**

To test this hypothesis, we sorted neuronal nuclei with normal and depleted TDP‐43 levels from human post‐mortem AD and Mild Cognitive Impairment (MCI) patients as well as Non‐neurological Disease (ND) Controls.

**Results:**

RNA sequencing results from these sorted nuclear isolates show substantial genome wide transcriptional alterations in gene expression as well as alternative splicing, and TDP‐43‐associated cryptic exon inclusion.

**Conclusions:**

These results support the significant role of TDP‐43 pathology on the transcriptome of up to half of AD patients and the need for further exploration of a common but overlooked aspect of the most common progressive dementia in patients today.

